# Signatures of Gate-Driven Out-of-Equilibrium Superconductivity in Ta/InAs Nanowires

**DOI:** 10.1021/acsnano.2c10877

**Published:** 2023-03-13

**Authors:** Tosson Elalaily, Martin Berke, Máté Kedves, Gergő Fülöp, Zoltán Scherübl, Thomas Kanne, Jesper Nygård, Péter Makk, Szabolcs Csonka

**Affiliations:** † Department of Physics, Institute of Physics, Budapest University of Technology and Economics, Müegyetem rkp. 3., H-1111 Budapest, Hungary; ‡ MTA-BME Superconducting Nanoelectronics Momentum Research Group, Müegyetem rkp. 3., H-1111 Budapest, Hungary; ¶ Department of Physics, Faculty of Science, Tanta University, Al-Geish St., 31527 Tanta, Gharbia, Egypt; § MTA-BME Correlated van der Waals Structures Momentum Research Group, Müegyetem rkp. 3., H-1111 Budapest, Hungary; ∥ Center for Quantum Devices and Nano-Science Center, Niels Bohr Institute, University of Copenhagen, Universitetsparken 5, DK-2100, Copenhagen, Denmark

**Keywords:** field effect, nanowire, gate-controlled supercurrent, hot electron injection, phonons, phase slips

## Abstract

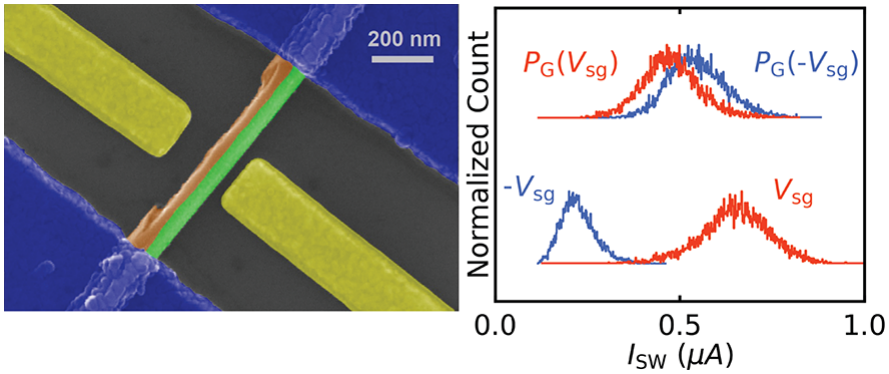

Understanding the microscopic origin of the gate-controlled supercurrent (GCS) in superconducting nanobridges is crucial for engineering superconducting switches suitable for a variety of electronic applications. The origin of GCS is controversial, and various mechanisms have been proposed to explain it. In this work, we have investigated the GCS in a Ta layer deposited on the surface of InAs nanowires. Comparison between switching current distributions at opposite gate polarities and between the gate dependence of two opposite side gates with different nanowire–gate spacings shows that the GCS is determined by the power dissipated by the gate leakage. We also found a substantial difference between the influence of the gate and elevated bath temperature on the magnetic field dependence of the supercurrent. Detailed analysis of the switching dynamics at high gate voltages shows that the device is driven into the multiple phase slips regime by high-energy fluctuations arising from the leakage current.

Since superconducting circuits have the potential to realize electronics with short switching time and ultralow power consumption, various architectures have been developed for integrating semiconductor technology with superconducting devices to reduce the high power consumption required for cooling the high-density semiconductor-based microchips.^1−3^ The cryotron,^4^ Josephson cryotron,^5^ rapid single flux quantum (RSFQ) device,^6^ and nanocryotron (nTron)^1^ were all developed as building blocks for superconducting switches; however, their scalability or even the difficulty of interfacing with CMOS electronics limited their applications. In recent years, suppression of supercurrent by applying a voltage to a gate electrode in the vicinity of a superconducting metallic nanowire has attracted much attention as a promising building block for highly scalable superconducting switches.^7−16,16−28^ In some works, the effect is attributed to the large electric field (10^8^ V/m) at the superconducting surface,^7−16,16−21^ which distorts the superconducting state and leads to the quenching of the superconductivity.^29−33^ Other studies^22−28^ reported a correlation between the gate-controlled supercurrent (GCS) and the leakage current flowing between the gate and the superconducting device. Some of these studies suggest that the GCS results from ballistic injection of high-energy quasiparticles.^24−26^ In another work, the quenching of the supercurrent was attributed to the absorption of phonons emitted in the relaxation process of high-energy electrons injected from the gate electrode.^28^ In order to engineer efficient superconducting switches for future electronic applications, it is important to understand the dominant mechanism behind the GCS effect.

In this work, we have studied the GCS in a superconducting Ta shell deposited on the surface of InAs nanowires.^34^ We chose Ta because of its strong spin–orbit interaction,^35,36^ so it is expected that the electric field has a strong influence on the superconducting state. We investigated the influence of the distance between the gate and the nanowire on the suppression of the supercurrent for the fabricated devices. Also, the magnetic field dependence of the supercurrent under the influence of the gate voltage and elevated temperatures was investigated. In addition, the switching current distribution at opposite gate polarities and at different current ramp speeds was studied. Furthermore, we give a detailed analysis for the switching dynamics at high gate voltages. Our findings contradict the proposed theoretical explanations based on electric fields or ballistic injection of high-energy electrons, and they are consistent with the nonequilibrium phonon picture as the origin of the GCS effect.

## Results and Discussion

**Figure 1:**
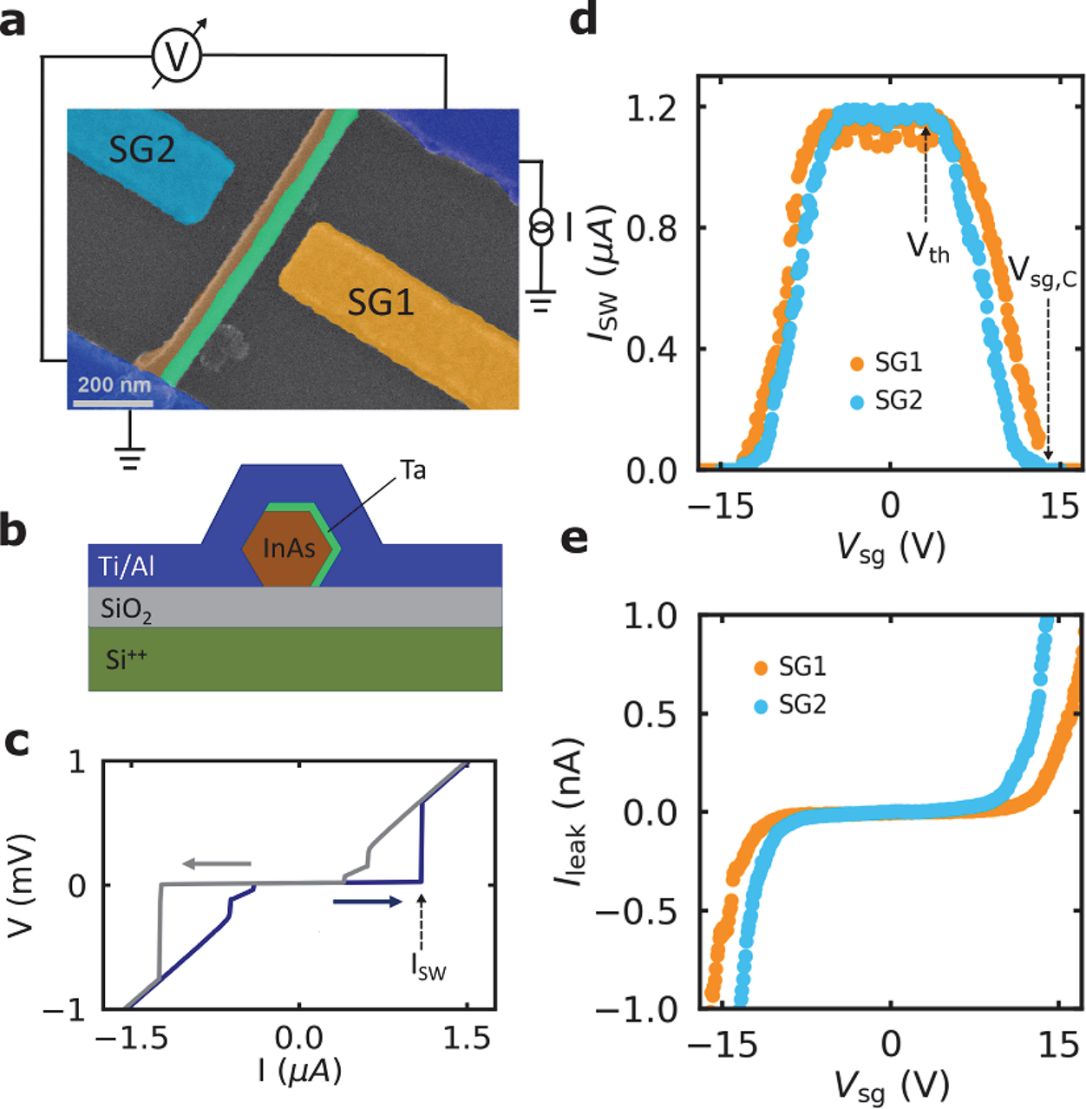
Device geometry and gate dependence characterization (device A). (a) A false-colored SEM image and (b) schematic of the side view of the nanowire device. (c) *I*–*V* characteristics of the device measured at 35 mK. As the bias current ramps from negative to positive values (blue arrow), the device switches to a finite-resistance state at the switching current *I*_SW_ = 1.17 μA. If the current ramps in the opposite direction (gray arrow), the device switches back to the superconducting state at two successive retrapping current values at ≃0.61 μA and ≃0.4 μA. (d) *I*_SW_ as a function of *V*_sg_ (at magnetic field *B* = 0.1 T) applied to SG1 (orange curve) and SG2 (light blue curve) with nanowire–gate spacings of ≃65 and ≃115 nm, respectively. (e) The leakage current as a function of *V*_sg_ for both gates.

**Figure 2:**
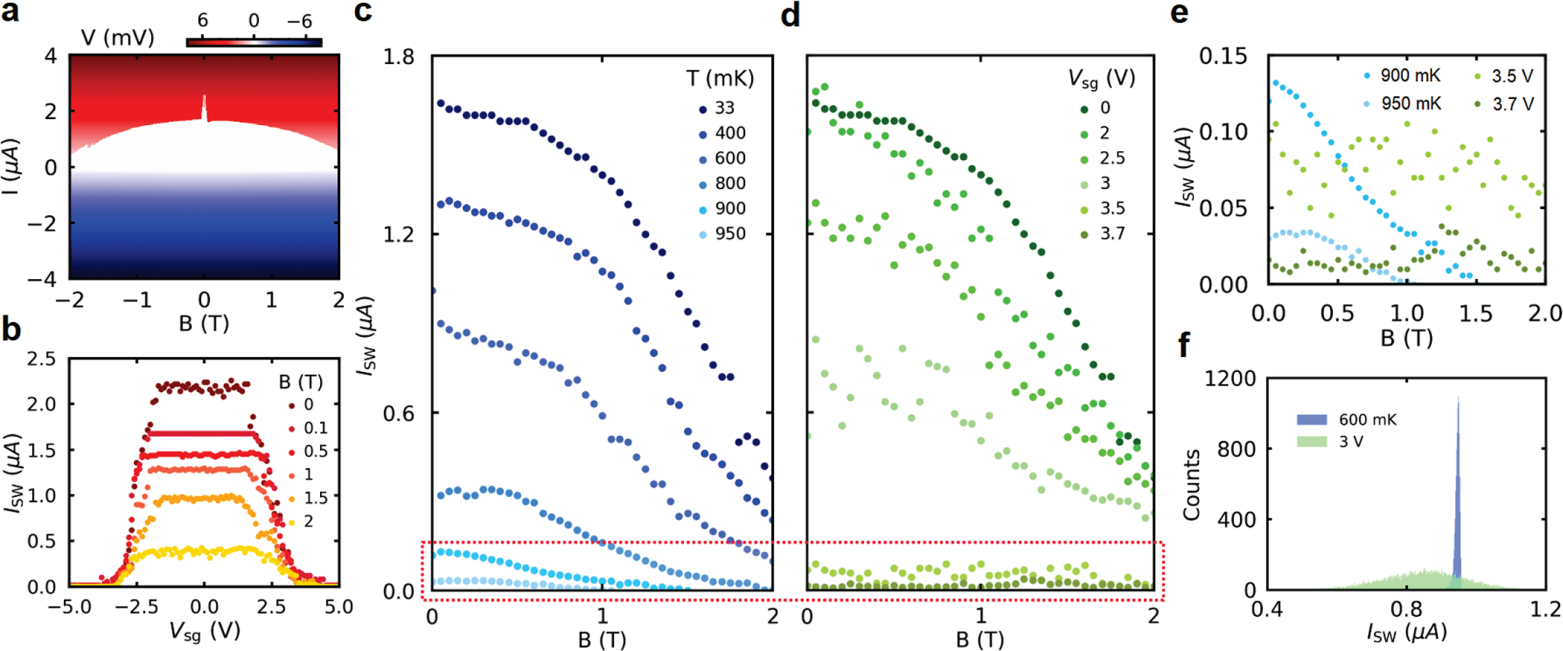
Magnetic field dependence and comparison between the GCS effect and effect of bath temperature (device B, *d* = 35 nm). (a) *I*–*V* curve as a function of out-of-plane magnetic field *B* up to ±2 T. (b) *I*_SW_ as a function of *V*_sg_ at various values of B-field up to 2 T. (c) *I*_SW_ as a function of the B-field at various elevated temperatures and (d) at various values of *V*_sg_. (e) Magnification of the curves surrounded by the red rectangle in (c) and (d). (f) Comparison between the SCDs measured at *T* = 600 mK (blue) and *V*_sg_ = 3 V (green).

**Figure 3:**
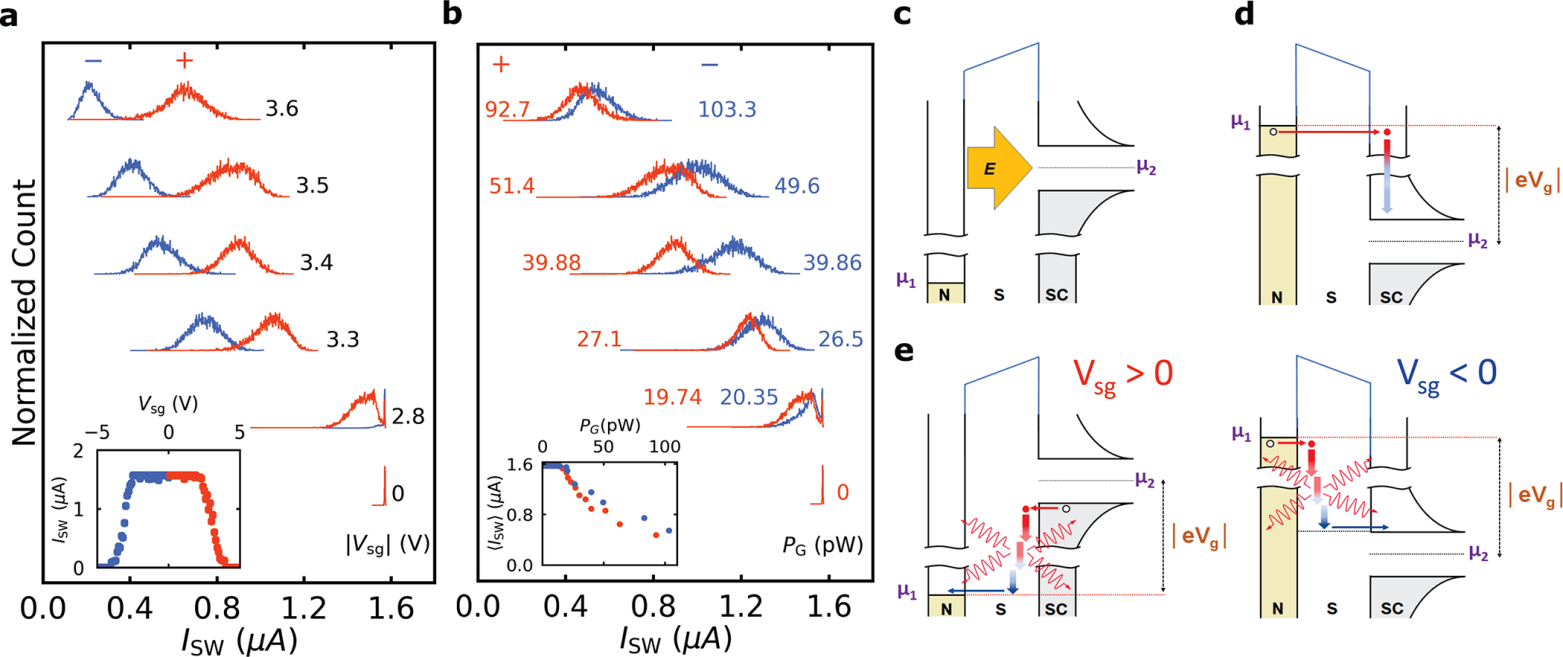
SCD measurements and schematics for different proposed mechanisms of the GCS effect (device C, *d* = 30 nm). (a) SCDs measured at positive (orange) and negative (blue) gate polarity and paired at the same |*V*_sg_|. The SCDs are normalized to their maximum counts and shifted on the *y*-axis for clarity. The inset shows *I*_SW_ as a function of *V*_sg_ for the investigated device measured at 0.1 T. (b) SCDs measured at positive (orange) and negative (blue) gate polarity and paired at approximately the same *P*_G_. The inset shows the mean value of ⟨*I*_SW_⟩ of SCDs measured at both gate polarities as a function of *P*_G_. (c) Schematic diagram of the electric field *E* applied from the metallic gate N to the superconducting nanowire SC at positive gate polarity. The colored/uncolored parts represent occupied/unoccupied states. (d) Schematic diagram of the ballistic electron injection from the gate to the nanowire at negative gate polarity. The high-energy electron (red circle) tunnels through the potential barrier of the substrate S and relaxes to the lowest unoccupied state (close to the superconducting gap edge), releasing heat on the SC side. (e) Schematic diagram of relaxation of high-energy electrons in the substrate when injected from the SC/N side to N/SC at positive/negative gate polarity in the left/right panels. In the case of positive gate polarity, the electrons relax close to the SC side (superconducting nanowire) so that it is heated more than in the case of negative gate polarity at the same *P*_G_.

**Figure 4:**
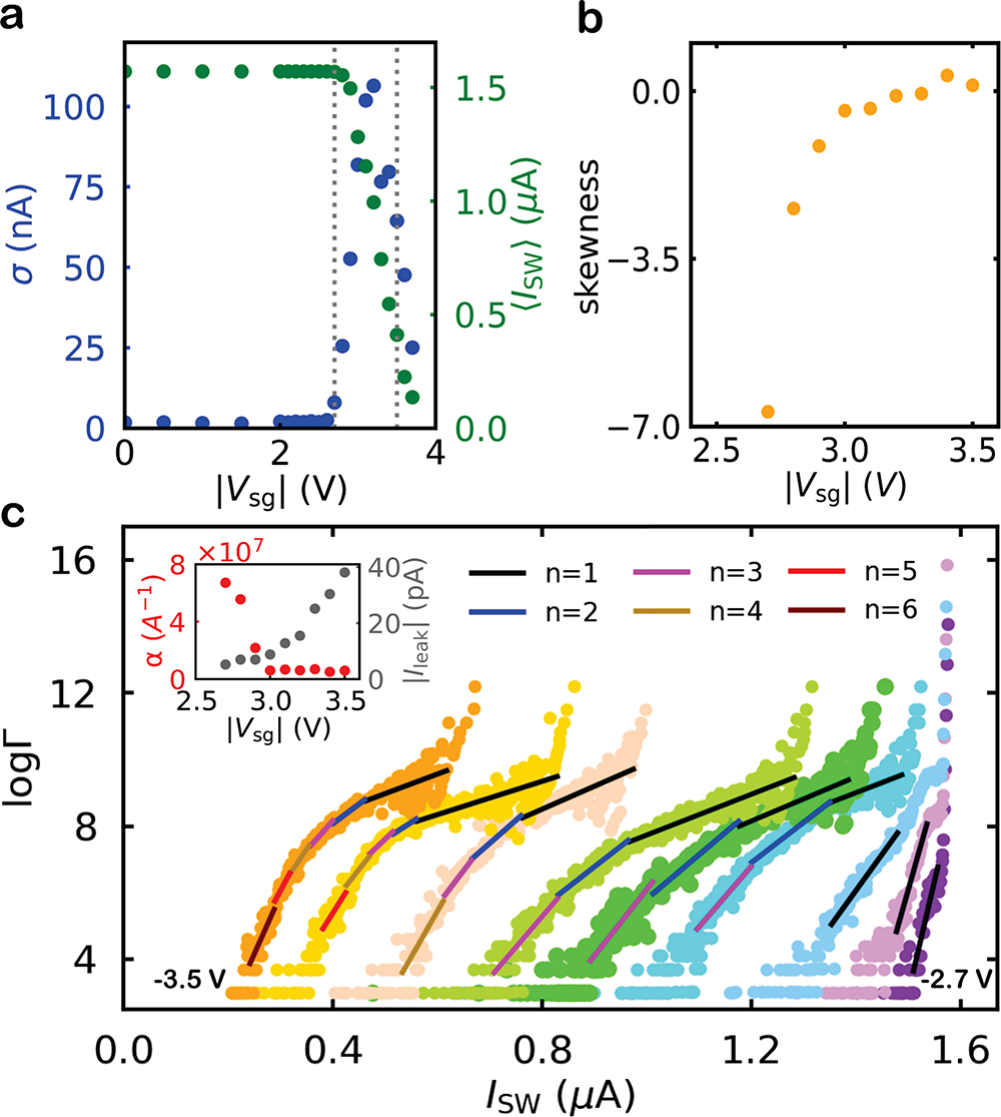
Analysis of the switching dynamics under the influence of *V*_sg_ (device C, *d* = 30 nm). (a) Standard deviation σ and mean value ⟨*I*_SW_⟩ as a function of |*V*_sg_| for all SCDs measured at negative gate polarity in the blue and green curves, respectively. (b) The calculated skewness as a function of |*V*_sg_| for SCDs measured with a step of 0.1 V in the interval [−3.5, −2.7 V] (surrounded by the vertical gray dotted lines in panel a) where a corresponding increase in *I*_leak_ is observed. (c) Logarithm of escape rate Γ as a function of *I*_SW_ (colored curves) for different values of *V*_sg_ from −3.5 V (orange curve) up to −2.7 V (purple curve) with a step of 0.1 V. The colored solid lines represent the fitting of different portions of these curves with an exponential of higher orders *n* of the slope α. The inset shows the variation of the slope α with increasing *V*_sg_ (red curve) and the corresponding *I*_leak_ as a function of *V*_sg_ (gray curve).

In our device configuration, we used InAs nanowires with a 20-nm-thick Ta shell layer deposited on only three facets of the nanowire.^34^ In order to investigate the impact of the gate on the supercurrent flowing in the Ta layer, four-terminal nanowire-based devices were fabricated with the configuration shown in Figure 1a,b. The Ta/InAs nanowires (green/brown) were deposited on a doped Si wafer with a 290-nm-thick oxide layer. Four Ti/Al contacts (blue) with a thickness of 10/80 nm were fabricated on the top of the nanowire with a distance of 1 μm for quasi-four-terminal measurements. Two metallic Ti/Au side gates, SG1 (orange) and SG2 (light blue), with a thickness of 7/33 nm were placed with unequal spacings and on opposite sides of the nanowire. This provides a possibility to study the GCS effect for the device with gates at different spacings. The results presented in this paper are based on measurements performed on three different devices, A, B, and C, with the same device geometry, but with different values of nanowire–gate spacing *d* in the range from 30 to 120 nm. The results in Figure 1 were measured on device A and in Figure 2 on device B, while the results in Figure 3 and their analysis in Figure 4 were performed on device C.

The current–voltage (*I*–*V*) characteristics measured at 35 mK show a clear switching from the superconducting state to the normal state at the switching current *I*_SW_ ≃ 1.17 μA (see blue curve in Figure 1c). When the measurements are carried out in the opposite sweep direction (gray curve), the device shows a hysteretic behavior and switches back to the superconducting state at two successive retrapping current values at ≃0.61 μA and 0.4 μA. This hysteretic behavior can be attributed to large Joule heating dissipated in the resistive state.^37^ The GCS is investigated by measuring the dependence of *I*_SW_ under the influence of gates SG1 (orange) and SG2 (light blue) with *d* of ≃65 and ≃115 nm, respectively. Figure 1d shows *I*_SW_ as a function of *V*_sg_ for both gates, where each of the plotted curves has the same color as the corresponding gate in Figure 1a. The plot reveals that both gates completely switch the device to the normal state at almost the same critical gate voltage, *V*_sg,C_ ≃ ±13 V. Even though the nanowire–gate spacing for SG1 is about half that for SG2, SG2 still suppresses *I*_SW_ at lower threshold gate voltage *V*_th_ than SG1. Importantly, at *V*_th_, a correspondingly large increase in the gate leakage current *I*_leak_ is observed for each of the gates (see Figure 1e), which has also been reported elsewhere.^22,23,25,26,28^

The dependence of the supercurrent in our device on the out-of-plane magnetic field *B* is shown in Figure 2a, where the *I*–*V* curves are measured as a function of the *B*-field up to ±2 T. The white region represents the zero-resistance state, with a transition to and from the normal state (red and blue regions) at the switching and retrapping current values in the positive and negative bias current values, respectively. The magnitude of *I*_SW_ shows a rapid suppression with increasing *B*-field below 100 mT and then slowly decreases with further increasing the magnetic field up to 2 T. The sharp decrease in the critical current below 100 mT coincides with the *B*_C_ of the Al electrodes contacting the nanowire;^23^ therefore we believe that this decrease is a result of the Al contacts switching to normal state. Although the maximum *B*-field in our setup (2 T) does not allow full suppression of the superconducting state in the Ta shell, based on the measured trend, *B*_C_ is expected to be about 3.5 T, which is consistent with earlier findings on identical Ta/InAs nanowires.^38^

The gate dependence of *I*_SW_ under the influence of the B-field is shown in Figure 2b. *I*_SW_ is plotted as a function of *V*_sg_ at different values of magnetic field up to 2 T. No significant change in *V*_sg,C_ with increasing *B*-field was observed, which is in contrast to the dependence observed for Ti and Al nanostructures.^7,23^Figure 2c and d show the dependence of *I*_SW_ on *B*-field under influence of temperature *T* and *V*_sg_, respectively. In the former case, *I*_SW_ decreases with increasing *T*, as expected, accompanied by a suppression of *B*_C_, giving *B*_C_ = 2 T at 800 mK and *B*_C_ = 1.5 T at 900 mK. In the case of the gate control, *I*_SW_ also decreases with increasing *V*_sg_, but surprisingly, *no change in B*_C_*was observed*. For a better comparison, Figure 2e shows a zoom-in of the curves in both dependencies marked by the red rectangle and having almost the same magnitude of *I*_SW_ (at *B* = 0 T). It can be clearly seen that the *B*_C_ dependence behaves differently under the influence of temperature and gate voltage. While from 900 mK to 950 mK *B*_C_ further decreases from 1.5 to 1 T, *I*_SW_ does not seem to be suppressed by the magnetic field in the case of the gate (see also the Supporting Information), in strong contradiction to other works in which a significant change of *B*_C_ with gate voltage was observed.^10,16,23^

Another noticeable difference between temperature and gate dependence is that *I*_SW_ exhibits large fluctuations at finite gate voltages (see green curves in Figure 2e). In order to investigate this effect, the switching current distribution (SCD) at finite temperatures and gate voltages is measured by ramping the current at constant speed from 0 to 3 μA for 10,000 times and recording the corresponding *I*_SW_ value every time (see Methods). A comparison between the SCDs obtained at 600 mK and 3 V is shown in Figure 2f. Despite the fact that both histograms have almost the same mean value ⟨*I*_SW_⟩, the width of the histogram obtained under influence of the gate voltage is an order of magnitude larger than that obtained at elevated bath temperature. The large gate-induced broadening is consistent with refs (14), (19), (22), (26), and (28) and shows that the gate voltage induces an out-of-equilibrium state in the superconducting nanowire, which cannot be described with an effective temperature.

In the following, we will compare the SCDs measured at positive and negative gate polarity, as they are expected to behave differently for different microscopic origins of the GCS. The dependence of *I*_SW_ on *V*_sg_ of the device is shown in the inset of Figure 3a, where the positive and negative gate polarities are represented by the orange and blue curves, respectively. Figure 3a shows the SCDs measured at the same |*V*_sg_| but with opposite polarities are paired and shifted along the *y*-axis for clarity. For simplicity, we made the measurements with the Al leads in the normal state, at *B* = 100 mT.^23^ There is a clear difference in the shape and ⟨*I*_SW_⟩ of SCDs paired at equal |*V*_sg_|. In addition, we also paired SCDs for opposite gate polarities and with approximately the same power dissipated at the gate *P*_G_ = *I*_leak_·*V*_sg_ as shown in Figure 3b. Comparing Figure 3a and b, one can conclude that the pairing at the same power gives a better match between SCDs with opposite polarities. We also found that the SCDs measured at positive polarity have a slightly smaller ⟨*I*_SW_⟩ than those measured at negative polarity at the same *P*_G_ (see inset in Figure 3b).

Assuming that the electric field *E* applied by the gate (Figure 3c) is responsible for the suppression of *I*_SW_,^29−33^ its effect should not depend on the sign of *E*. Therefore, we expect the SCD obtained at a given voltage *V*_sg_ to be identical to the SCD obtained at the same gate voltage with the opposite sign, −*V*_sg_. Since the measured SCDs do not match at opposite polarities (see Figure 3a), our results contradict the electric field-based explanation. Another possible microscopic picture is that the CGS is caused by ballistic injection of high-energy quasiparticles, as shown in Figure 3d. After injection of these electrons, their energy is released by relaxation, heating the side on which they end up. Therefore, for negative gate polarity (Figure 3d), they heat the superconducting bridge, while for positive polarity they heat the gate electrode instead. Thus, a stronger suppression of superconductivity is expected for negative polarity. Therefore, at the same *P*_G_ value, the mean value of the distribution is expected to be significantly smaller for negative polarity than for positive polarity. Comparing this prediction with the measured results in Figure 3b, one can conclude that the experimental findings are just opposite, so that ballistic injection of electrons can also be excluded.

The most likely explanation for our results is the generation of phonons by a series of relaxation events of the high-energy electrons in the substrate.^28^ The small shift between the ⟨*I*_SW_⟩ measured for the two polarities (see Figure 3b) can be attributed to the short energy relaxation length of electrons in SiO_2_ (≤3 nm) at high electric fields compared to nanowire–gate spacing (*d* = 30 nm).^39−42^ Thus, at positive gate polarity, it is expected that the high-energy electrons will relax close to the nanowire (Figure 3e, left panel), and the generated phonons can heat the superconducting nanowire more than at negative gate polarity (Figure 3e, right panel).

The standard deviation σ of SCDs measured under the influence of the gate is represented by the blue curve in Figure 4a. For small values of |*V*_sg_|, where *I*_leak_ is negligible, σ is independent of |*V*_sg_| and no significant change in the ⟨*I*_SW_⟩ of SCDs (green curve) was observed. Beyond *V*_th_ at |*V*_sg_| = 2.7 V, σ increases with |*V*_sg_| because the fluctuations assisted by *I*_leak_ become stronger and more frequent. This increases the probability of nanowire switching at small *I*_SW_ values with a corresponding suppression in the ⟨*I*_SW_⟩ of the SCD. This increase in the width of the SCDs is analogous to the typical temperature dependence (see the Supporting Information)^14,19,43^ associated with thermally activated phase slips.^44,45^ However, the large width of the SCDs obtained under the influence of the gate indicates that the system is driven to a nonequilibrium state where the fluctuations are an order of magnitude larger than expected from the bath temperature. With further increasing |*V*_sg_|, σ decreases and the SCDs become more symmetric, as shown by their calculated skewness in Figure 4b. This is analogous with the picture that the switching of the system is due to multiple phase slips (MPS) found at finite temperatures.^43^

Interestingly, the SCD in Figure 3a at *V*_sg_ = 2.8 V (orange curve) shows two peaks, a sharp one at 1.57 μA and a broad one around 1.5 μA. This distribution looks like the sum of two overlapping probability distributions, similar to distributions shown in refs (14) and (22). Since the probability distribution in this transition region depends strongly on the ramp speed of the bias current ν_I_ (see the Supporting Information), we could completely switch between the two distributions when the ramp speed was changed from 300 (at which the SCDs in Figure 3 are measured) to 9.375 μA/s. For a more accurate evaluation, it is better to transform the measured probability distributions into the speed-independent escape rate Γ(*I*, *T*) (see the Supporting Information) by the direct Kurkijärvi–Fulton–Dunkleberger (KFD) transformation:^45−47^
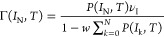
1where *w* is the bin size in the current axis of the measured probability distribution *P*(*I*, *T*), and *P*(*I*_k_, *T*) is the switching probability in the bias current interval [*kw*, (*k* + 1)*w*] with *k* ∈ [0, *N*]. Γ(*I*_N_, *T*) represents the rate at which the superconducting order parameter reaches zero under the influence of external fluctuations. Figure 4c shows the logarithm of the calculated Γ(*I*_N_, *T*) as a function of current for SCDs measured under the influence of *V*_sg_ in the interval [−3.5, −2.7 V]. As long as *V*_sg_ is small, the SCDs have a sharp peak around *I*_SW_ = 1.57 μA, resulting in a large escape rate around this value, which represents the escape rate due to quantum tunneling or thermal escape Γ_T_. As the influence of the gate voltage sets in (see, e.g., purple curve), a finite escape rate appears at lower *I*_SW_ values, corresponding to the gate-assisted escape rate Γ_L_. The latter contribution becomes the dominant escape rate at higher gate voltages (see, e.g., green curve).

For *V*_sg_ > −3 V, where *I*_leak_ is negligible (the first three curves from the right), Γ_L_ can be well fitted with an exponential curve (black solid line) given by
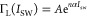
2with *n* = 1 and using α and *A* as fitting parameters. The switching dynamics in this region have been extensively studied in detail in ref (22). In this regime, the fluctuation events triggered by the gate were assumed to be rare and independent, and the dependence of the escape rate on the current is fitted by a single exponential. For *V*_sg_ ≤ −3 V, Γ_L_ deviates from the single-exponential dependence described by eq 2. For example, the light green curve measured at −3.2 V can be fitted at large current values using eq 2 with *n* = 1 (see black solid lines). Interestingly, the measured curve for *I*_SW_ < 0.9 μA can be well fitted by adding extra higher order terms with *n* = 2, 3, ..., keeping the same values of the fitting parameters. Further increasing *V*_sg_ (*I*_leak_) requires more higher order terms to fit the escape rate dependence (e.g., orange curve).^43^ The value of α required to fit the escape rate dependence decreases sharply as *V*_sg_ increases, and saturates at large values of *V*_sg_ (*I*_leak_) as shown in the red curve in the inset of Figure 4c, while the corresponding *I*_leak_ is shown in the gray curve.

The deviation of the escape rate dependence with current from a pure exponential at higher gate voltages is similar to elevated temperatures in ref (43). This can be attributed to the reduced impact for a single fluctuation event triggered by the leakage current, since the dissipation during the induced phase slip event is smaller at lower values of *I*_SW_.^43,45^ Thus, several coincident fluctuation events with corresponding induced MPS are required to trigger the switching of the nanowire to the normal state.^43,48,49^ In this regime, at large bias current values, the dissipation of a single MPS event (*n* = 1) is sufficient to switch the nanowire into the resistive state. On the other hand, at lower current values, the dissipation of a single MPS event is reduced and higher orders (*n* = 2, 3, ...) of the MPS event are required to trigger the resistive switching of the nanowire.^43^

In the following, we will compare our experimental results with the possible microscopic pictures. Starting from the two gates (Figure 1d,e), despite SG2 having almost twice the nanowire–gate spacing of SG1, it suppresses the *I*_SW_ at lower *V*_th_ than SG1. This contradicts the electric field picture as a possible explanation for the origin of the GCS. On the other hand, *I*_SW_ starts to be suppressed with the onset of leakage current between the nanowire and each of the gates (see the Supporting Information). In another cool-down, the influence of the two gates for the same device shows an opposite situation, as SG1 shows a stronger influence on *I*_SW_ than SG2 (see the Supporting Information). This excludes any concerns arising from the quite large dielectric constant of the InAs nanowire between SG2 and the Ta shell (see Figure 1a), which may lead to a larger influence of SG2 on *I*_SW_ than SG1. Interestingly, we found that the influence of the two gates on *I*_SW_ gives better matching with *P*_G_ in the two cool-downs (see the Supporting Information).

Accepting that the leakage current plays a key role in the GCS, a simple explanation arises: that the leaking electrons increase the temperature of the superconducting nanowire. We have investigated the *B*-field dependence of a superconducting nanowire with normal contacts, which allows efficient cooling of the superconducting nanowire. The *B*-field dependence at finite *T* and finite gate voltage was strictly different, indicating that the effect of leakage current cannot be described by a simple hot electron regime induced by elevated bath temperature. The highly nonequilibrium state of the superconductor at finite gate voltage is further supported by the broad SCDs in our work and in previous results.^14,19,22,26,28^ Our detailed comparison of the SCDs for different gate polarities (Figure 3) provided another important finding which is inconsistent with electric-field-induced suppression of superconductivity. Pairing of SCDs measured at opposite gate polarities at the same leakage current dissipation, *P*_G_, provided a better matching than at the same |*V*_sg_| (Figure 3a,b). This reveals that the suppression of *I*_SW_ depends not only on the energy of the injected electrons (e*V*_sg_) or the rate of their injection (*I*_leak_/e) but on the power dissipated at the gate, *P*_G_. Based on the ⟨*I*_SW_⟩ of the SCDs for the two polarities, the ballistic injection of electrons from the gate into the superconducting nanowire can be discarded. We conclude that the phonon-mediated excitation of the superconductor remains a microscopic picture consistent with the measured results.

Furthermore, we also noticed that the power dissipation at the gate required to fully suppress *I*_SW_, *P*_G,C_, is comparable to the power dissipation that occurs when the device switches to the resistive state, *P*_n_ = . For example, for device A, in the case of SG1 (the closer to the Ta shell), *P*_G,C_ ≃ 1.5 nW (see the Supporting Information), while *P*_n_ ≃ 1 nW (using *R*_n_ = 780 Ω and *I*_SW_ ≃ 1.17 μA).

Finally, a very large leakage current was required to quench *I*_SW_ when we investigated the GCS in similar Ta/InAs devices fabricated on a sapphire substrate (see the Supporting Information). These results indicate that the GCS depends mainly on the properties of the substrate and the leakage pathway between the gate and nanowire.

## Conclusions

We investigated the origin of GCS in the Ta half-shell layer deposited on InAs nanowires by various measurements. Devices with small nanowire–gate spacing (specifically devices B and C) fully switch to the normal state below *V*_sg_ = ±5 V, which makes them promising for integration into classical electronic circuits. When the wire is connected by electrodes in the normal state, the critical magnetic field *B*_C_ is not suppressed under the influence of the gate as for elevated temperatures. Moreover, the comparison of the switching current distributions at opposite gate polarities, as well as the gate dependence of two opposite side gates at different nanowire–gate spacings, shows that the power dissipated at the gate (*P*_G_) is the relevant parameter for this effect. Analysis of the switching dynamics under strong gate influence shows a deviation in the escape rate dependence with the bias current from a pure exponential. This indicates that the device is driven into the MPS regime by the high-energy fluctuations originating from the leakage current. The measurements on our devices are consistent with the nonequilibrium superconducting state resulting from the absorption of phonons generated by the leakage current and contradict the microscopic pictures proposing electric fields or ballistic injection of high-energy electrons as the origin of the GCS effect. GCS is a robust effect; it has been reported so far in very different circumstances: using various substrates and superconductors, different geometries, and even in suspended nanobridges^13^ or ionic gating.^21^ Since many of these measurements were done under very different experimental conditions, it is hard to make a direct comparison between the experiments. Furthermore, it is not obvious that a single mechanism should be expected to be responsible for all the measurements in the literature. Therefore, further investigations are required to reach a solid understanding of the contribution of different microscopic processes, which is essential to the use of GCS in future applications.

## Methods

InAs nanowires were grown by the VLS mechanism using molecular beam epitaxy and the Ta shell was deposited in situ under UHV using electron beam evaporation at a substrate temperature of about 25 °C. Based on the TEM characterization, the morphology of the Ta shells was continuous but granular on the InAs nanowires and was found to be noncrystalline.^38^

The Ta/InAs nanowires with a total diameter of ≃100 nm were deposited on the top of a doped Si wafer with a 290-nm-thick SiO_2_ layer by means of a hydraulic micromanipulator along with a high-magnification optical microscope. The nanowire device was fabricated in two separate electron beam lithography (EBL) steps. In the first step, four Ti/Al contacts with a thickness of 10/80 nm were fabricated. Prior to the metal evaporation, Ta/InAs nanowires were exposed to Ar-ion plasma milling for 8 min at 50 W to remove any oxides on the top of the Ta shell. In the second step, two metallic gates of Ti/Au layers with a thickness of 7/33 nm were fabricated with unequal spacing and on opposite sides of the nanowire. The metallic gates were fabricated in a separate lithography step, since a thin resist is used for precise alignment of the gates from the nanowire. Table 1 shows the dimensions of the investigated nanowire devices where *L*_NW_ is the nanowire segment length and *d*_1_ and *d*_2_ are the nanowire–gate spacings of gates SG1 and SG2, respectively.

**Table 1:** Dimensions of the Investigated Nanowire Devices

device	*L*_NW_ (μm)	*d*_1_ (nm)	*d*_2_ (nm)
A	1	65	115
B	1	35	115
C	1	30	120

The *I*–*V* characteristics of the device were measured by a pure DC measurement using a quasi-four-probe method in which the current was injected through the nanowire via a pair of Al contacts by using a standard voltage source (Basel DAC SP 927) with a series resistor of 1 MΩ, while the voltage was measured across the other pair with a differential voltage amplifier and a digital multimeter (Keithley 2001). The leakage current was recorded by measuring the voltage across a 10 MΩ preresistor connected to the gate and corrected according to the method reported in ref (23).

The SCD was measured using an NI-DAQ card (USB-6341), where a periodic current wave signal was engineered. This signal is composed of a positive linear ramp with an amplitude of 3 μA and a slope in the range from 9.375 to 300 μA/s followed by a 2.5 ms zero-current plateau for cooling down the superconducting device. This signal is repeated 10,000 times, and *I*_SW_ is extracted each time. All SCDs are measured at 0.1 T to switch the Al leads to the normal state. The skewness is calculated from the measured SCDs as
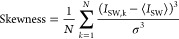
3where ⟨*I*_SW_⟩ and σ are the mean value and standard deviation of the SCD. All measurements were carried out in a Leiden Cryogenics CF-400 top-loading cryo-free dilution refrigerator system with a base temperature of 30 mK.

## Supporting Information Available

Gate dependence of the supercurrent for devices B and C, magnetic field dependence under the influence of the gate, temperature dependence of SCD, dependence of SCD and escape rate on the current ramp speed, comparison between the influence of two opposite side gates for device A, investigation of device A in another cool-down, dual-gate measurement, and measurements on different substrates (PDF)
