# Supplementary material for: Signatures of Gate-Driven Out-of-Equilibrium Superconductivity in Ta/InAs Nanowires
Source: ACS Nano. 2023 Mar 13;17(6):5528–35. doi: 10.1021/acsnano.2c10877 (PMC10062030; doi:10.1021/acsnano.2c10877)
Supplement: Supplementary file 1 — nn2c10877_si_002.pdf [file nn2c10877_si_002.pdf]

Signatures of gate-driven out of equilibrium superconductivity in Ta/InAs nanowires

Tosson Elalaily,[1,2,3] Martin Berke,[1,2] Máté Kedves,[1,4] Gergő Fülöp,[1,2] Zoltán Scherübl,[1,2] Thomas Kanne,[5] Jesper Nygård,[5] Péter Makk,[1,4,*] and Szabolcs Csonka[1,2,†]

[1]*Department of Physics, Institute of Physics, Budapest University of Technology and Economics, Müegyetem rkp. 3., H-1111 Budapest, Hungary*

[2]*MTA-BME Superconducting Nanoelectronics Momentum Research Group, Müegyetem rkp. 3., H-1111 Budapest, Hungary*

[3]*Department of Physics , Faculty of Science, Tanta University, Al-Geish St., 31527 Tanta, Gharbia, Egypt*

[4]*MTA-BME Correlated van der Waals Structures Momentum Research Group, Müegyetem rkp. 3., H-1111 Budapest, Hungary*

[5]*Center for Quantum Devices and Nano-Science Center, Niels Bohr Institute, University of Copenhagen, Universitetsparken 5, DK-2100, Copenhagen, Denmark*

I. GATE DEPENDENCE OF THE SUPERCURRENT FOR DEVICES B AND C

SFig. 1a and b show the I_{SW} as a function of V_{sg} for devices B and C, respectively. The gate dependence of the devices was investigated by using SG1 (the closest to Ta shell) at $B = 0.1$ T. Panels c and d show the corresponding I_{leak} as a function of V_{sg}.

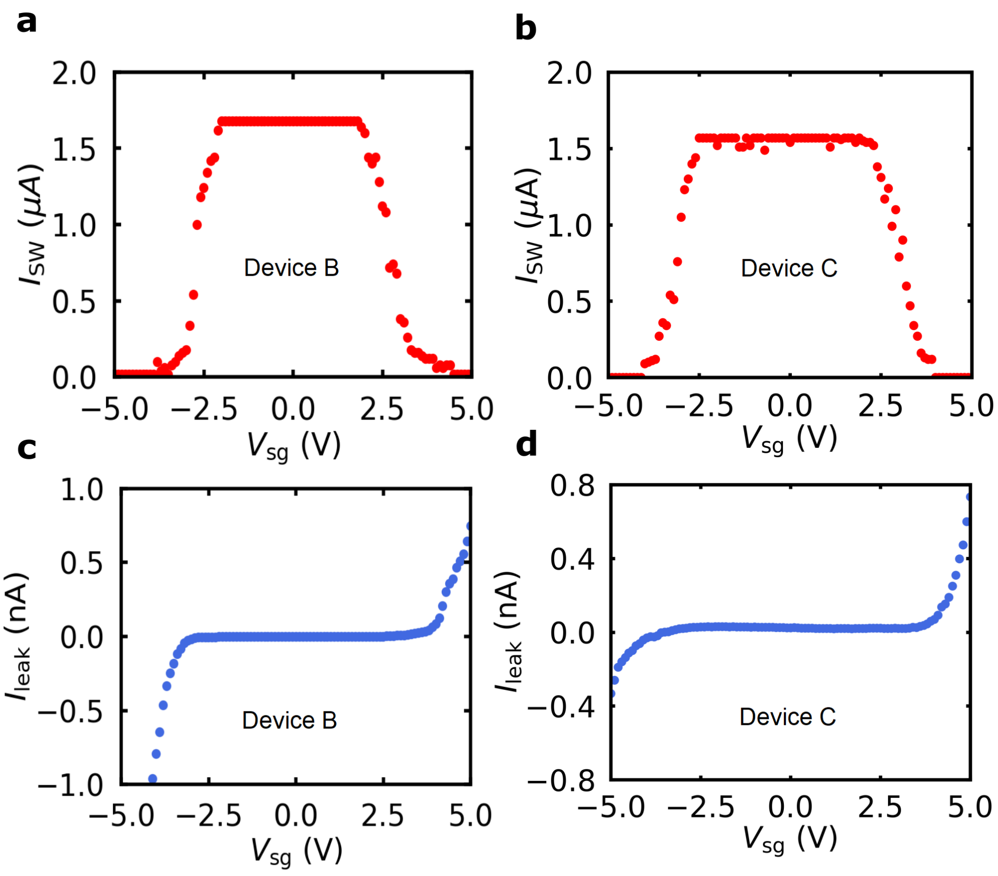

SFig. 1. **a**,**b** I_{SW} as a function of V_{sg} for devices B and C at $B = 0.1$ T and their corresponding leakage current in **c**,**d**, respectively.

II. MAGNETIC FIELD DEPENDENCE UNDER INFLUENCE OF THE GATE

In the main text (see Fig. 2e), we compared the B-field dependence of the supercurrent of device B at finite temperature and finite gate voltage (with similar I_{SW} at $B = 0$ T). We found that B_C decreases with increasing temperature, but not with increasing V_{sg}. SFig. 2a,b shows some selected (I-V) curves at different B-field values ≥ 1 T at $V_{sg} = 3.5$ V and 3.7 V, respectively, separated in the x-axis for clarity.

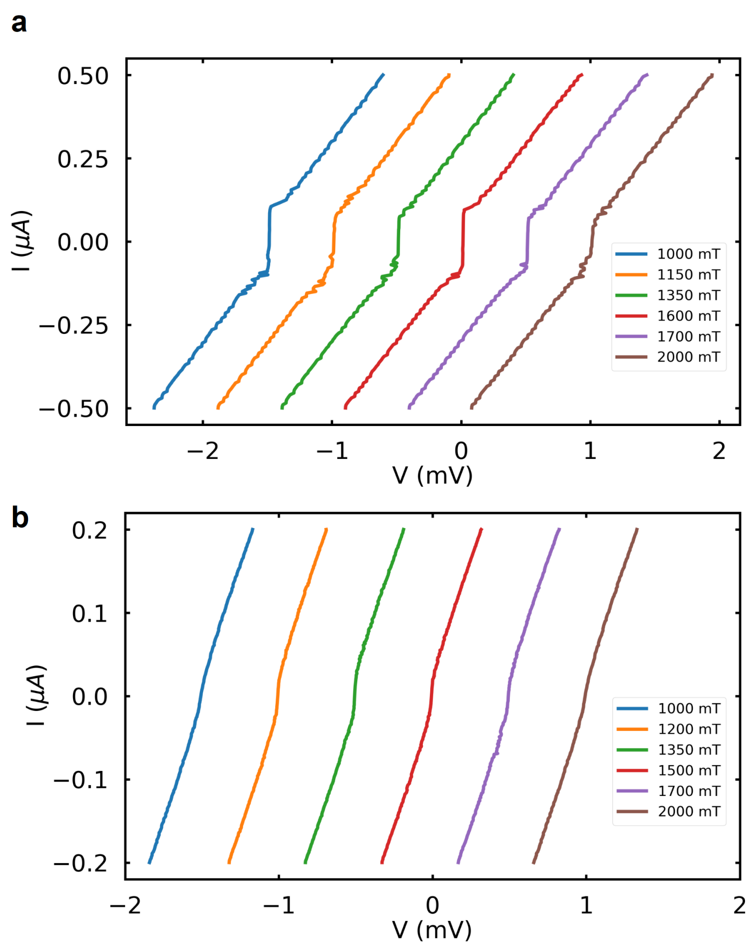

SFig. 2. **a** Selected (I-V) curves at different B-field values ≥ 1 T at $V_{\mathrm{sg}} = 3.5$ V and **b** at $V_{\mathrm{sg}} = 3.7$ V.

III. TEMPERATURE DEPENDENCE OF SCD

The dependence of SCDs measured at elevated temperatures for device B is shown in SFig. 3, in which SCDs measured at different temperatures at $B = 0.1$ T and normalized to their maximum count are plotted and separated on the y-axis for clarity. The corresponding standard deviation σ and mean value $\langle I_{\mathrm{SW}} \rangle$ are plotted as a function of temperature T in the top left and bottom right insets, respectively.

The standard deviation σ follows the expected conventional dependence as a function of T (see Refs. 1,2), which can be divided into three distinct regions (different colored regions in the upper left inset) where the switching mechanism is attributed to quantum phase slips (QPS), thermally activated phase slips (TAPS), and multiple phase slips (MPS). Saturation of the width at the lowest temperatures can also originate from non- ideal thermalization of

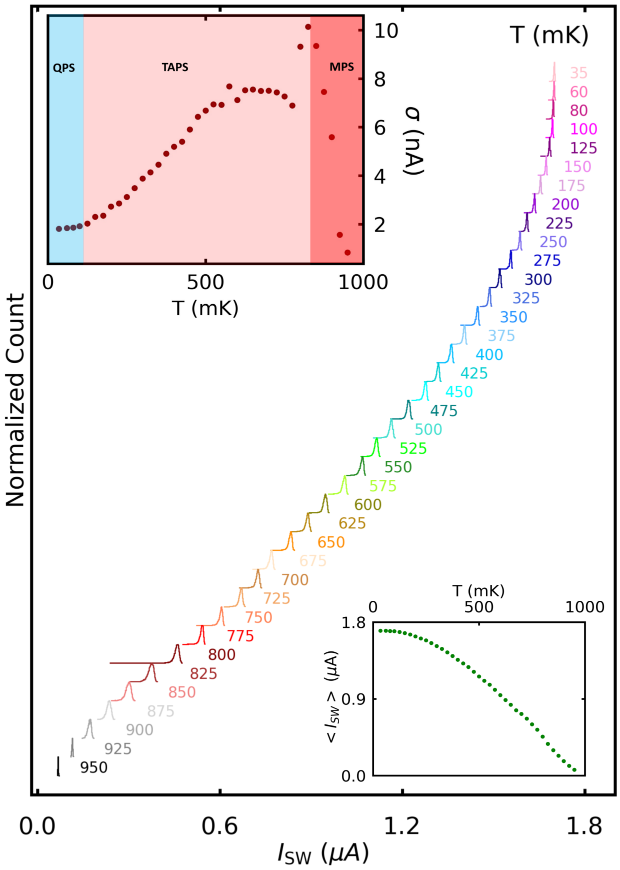

SFig. 3. Temperature dependence of SCDs for device C. In the top left, the standard deviation σ of the SCDs is plotted as a function of temperature. The colored regions represent the quantum phase slips (QPS) regime (light blue), the thermally activated phase slips (TAPS) regime (pink), and the multiple phase slips (MPS) regime (orange-red). In the bottom right inset, the $\langle I_{\rm SW} \rangle$ of the SCDs is plotted as a function of temperature T.

the electron bath, in which case this could still be part of the TAPS regime. Since this will not play an important role in our analysis, we will refer to this regime as the QPS regime for simplicity.

IV. DEPENDENCE OF SCD AND ESCAPE RATE ON THE CURRENT RAMP SPEED

In this section, we discuss the dependence of SCDs on the current ramp speed $\nu_{\rm I}$ for device C in another cool-down in which the gate dependence of $I_{\rm SW}$ is changed ($V_{\rm th}$ and

$V_{sg,C}$) compared to that in the main text as shown in the inset of SFig. 4b. SFig. 4a shows the SCDs measured at three different values of V_{sg}, and for each value they are measured at six different values of the current ramp speed ν_I. The values of V_{sg} are chosen so that the measured SCDs are at the beginning (0 V), within (1.35 V), and at the end (1.6 V) of the transition region between quantum tunneling (or thermal activation) and leakage-assisted phase slips.

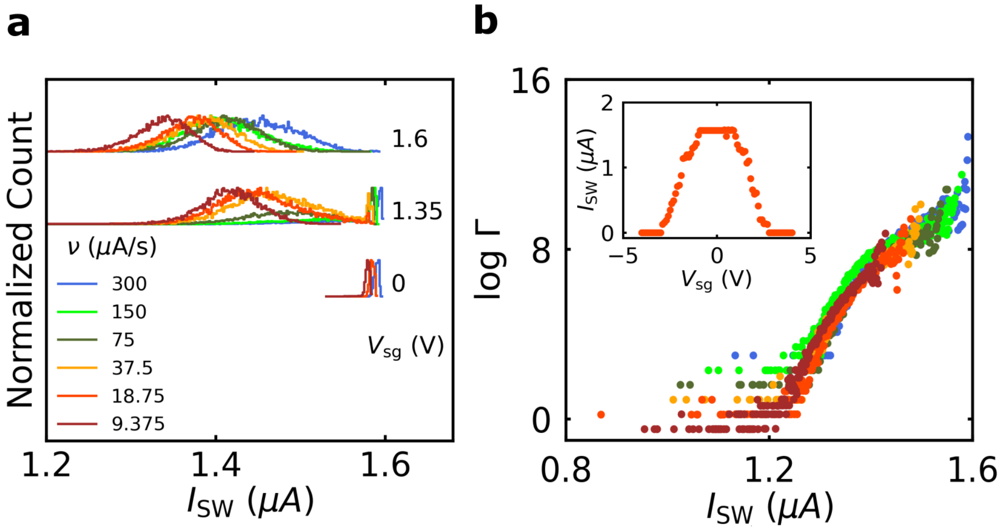

SFig. 4. **a** For device C , the SCDs were measured at V_{sg} =0, 1.35, 1.6 V, and for each value they were measured at different current ramp speeds ν_I. The SCDs are normalized by their maximum number of counts and separated on the y-axis. **b** Logarithm of escape rate Γ as a function of I_{SW} at different current ramp speeds ν_I for SCDs measured at V_{sg} = 1.6 V. The inset shows I_{SW} as a function of V_{sg} for the device.

At infinitely high values of ν, the superconductor is expected to have no time to switch prematurely in the presence of external fluctuations, and it will switch at $I_{SW} = I_C$, at which the system prefers to set at the lower energy of the normal state[2]. Thus, when ν_I increases from 9.375 to 300 μA/s, the $\langle I_{SW}\rangle$ of the histograms shifts to higher current values, as shown in SFig. 4a. Interestingly, the SCD e.g. at 1.35 V appears as two overlapping probability distributions, where a complete switch between the two distributions depends on the value of ν_I. Despite the SCDs depend strongly on ν_I, the transformation of the measured probability distributions into the escape rate $\Gamma(I, T)$ is independent of ν_I[2]. The transformed $\Gamma(I, T)$ for V_{sg} = 1.6 V evaluated from SCDs with different speeds are plotted in SFig. 4b.

V. COMPARISON BETWEEN THE INFLUENCE OF TWO OPPOSITE SIDE GATES FOR DEVICE A

Fig.1d and e in the main text show the dependence of I_{SW} on V_{sg} and the corresponding I_{leak} as a function of V_{sg} for two opposite side gates (SG1 and SG2) for device A. From these data, a parametric curve is plotted between I_{SW} and I_{leak} for the two gates in SFig. 5a. The plot shows that I_{SW} is suppressed for both gates at the onset of I_{leak}, despite it decreases at different V_{th}. A larger I_{leak} is required to switch the device to the normal state at negative gate polarity than at the opposite polarity. Plotting I_{SW} as a function of P_G as shown in SFig. 5b, a better matching between the two curves is observed. We also noticed that for SG1, $P_{G,C}$ is around 1.5 nW which is comparable to the switching power of the device $P_n \simeq$ 1 nW.

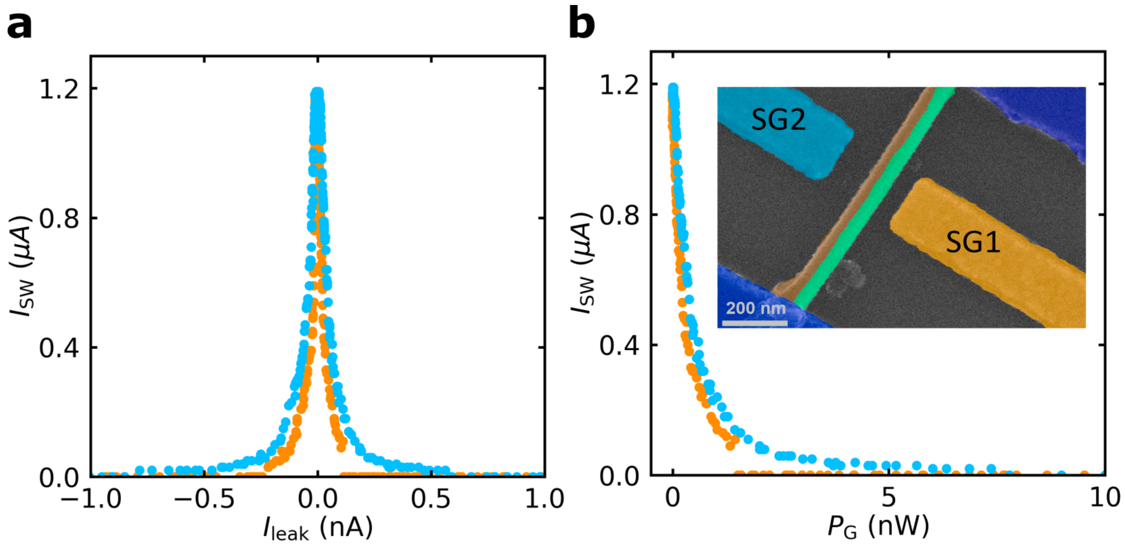

SFig. 5. **a** Parametric curve between I_{SW} and I_{leak} for SG1 (orange curve) and for SG2 (light blue) of device A. **b** I_{SW} as a function of P_G for both gates. The inset show a false colored SEM image for the investigated device with two opposite side gates colored same as their corresponding curves.

VI. INVESTIGATION OF DEVICE A IN ANOTHER COOLDOWN

By Investigating device A in another cool-down, the gate dependence of I_{SW} (see SFig. 6a) under the influence of SG1 (orange curve) and SG2 (light curve) gives an opposite situation to that obtained in the first cool-down (see Fig.1d,e in the main text). In this cool-down,

SG1 suppresses I_{SW} at lower V_{th} and $V_{\mathrm{sg,C}}$ than SG2. On the other hand, a corresponding increase in I_{leak} is observed at V_{th} for the two gates (see SFig. 6b).

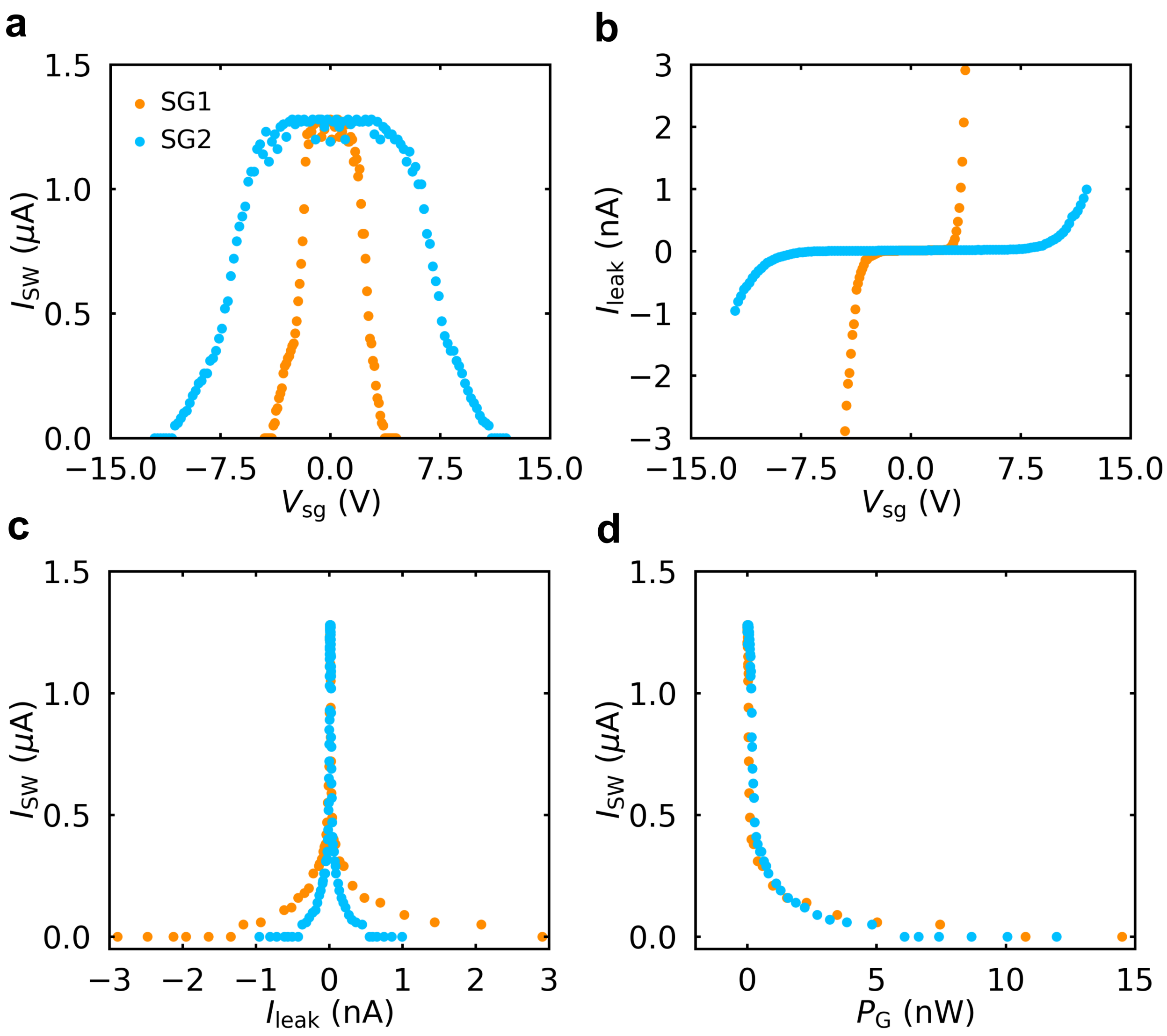

SFig. 6. **a** I_{SW} as a function of V_{sg} for SG1 (orange curve) and for SG2 (light blue curve) of device A in another cool-down.**b** I_{leak} as a function of V_{sg} for both gates. **c** Parametric curve between I_{SW} and I_{leak}. **b** I_{SW} as a function of P_{G} for both gates.

As in the previous section, plotting a parametric curve between I_{SW} and I_{leak} (see SFig. 6c) for the two gates shows that I_{SW} is suppressed at the onset of I_{leak}. Moreover, a strong matching between the influence of the two gates is obtained when I_{SW} is plotted as a function of P_{G}, as shown in SFig. 6d.

VII. DUAL-GATE MEASUREMENT

In this section, we will examine the effect of the two opposite side gates on the suppression of I_{SW} as well as on $V_{\mathrm{sg,C}}$. SFig. 7a shows I_{SW} as a function of V_{sg2} at different values of V_{sg1}. For $V_{\mathrm{sg2}} = 0$ V, I_{SW} decreases as expected with increasing V_{sg1}, while with increasing V_{sg2}, the dependence of I_{SW} on V_{sg2} looks quite similar for all values of V_{sg1} and no change in $V_{\mathrm{sg2,C}}$ is observed. The independence $V_{\mathrm{sg,C}}$ for one of the gates from the influence of the other has already been reported in Ref. 3, where the electric field was proposed as the origin of the gating effect. However, in our case a corresponding increase in the leakage current was observed as shown SFig. 7b.

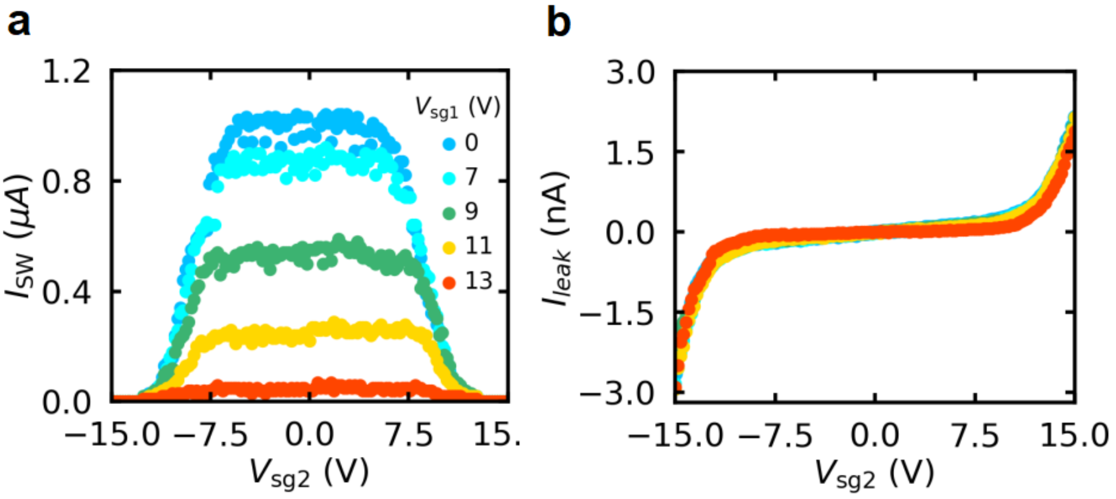

SFig. 7. **a** I_{SW} and I_{leak} as a function of V_{sg2} at different values of V_{sg1} for device A **b** The corresponding V_{sg} versus I_{leak}.

VIII. MEASUREMENTS ON DIFFERENT SUBSTRATE

We have investigated the GCS in another Ta/InAs nanowire device with the same device configurations but fabricated on a sapphire substrate. Despite I_{SW} is not fully suppressed by V_{sg} in the ± 17 V window (see SFig. 8a), the corresponding I_{leak} (see SFig. 8b) and thus P_{G} is two orders of magnitude higher than for the devices fabricated on Si^{++}/SiO_2 substrate shown in the main text. A possible reason could be that in case of a phonon mediated scenario the different type of the substrates allow different phonon generation.

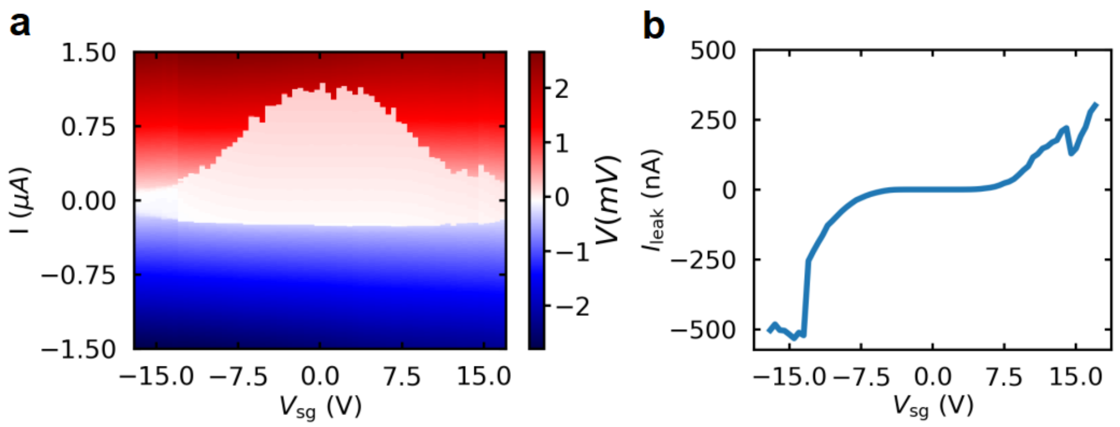

SFig. 8. **a** $I-V$ curve as a function of $\pm V_{sg}$ of a Ta/InAs nanowire device fabricated on a sappire substrate. **b** The corresponding I_{leak} as a function of $\pm V_{sg}$.

---

* makk.peter@ttk.bme.hu

† szabolcs.csonka@ttk.bme.hu

[1] D. McCumber and B. Halperin, Physical Review B **1**, 1054 (1970).

[2] A. Bezryadin, *Superconductivity in Nanowires: Fabrication and Quantum Transport* (John Wiley & Sons, Weinheim, Germany, 2013).

[3] F. Paolucci, G. De Simoni, P. Solinas, E. Strambini, N. Ligato, P. Virtanen, A. Braggio, and F. Giazotto, Physical Review Applied **11**, 024061 (2019).
